# Case Report: Sustained Remission Due to PD-1-Inhibition in a Metastatic Melanoma Patient With Depleted B Cells

**DOI:** 10.3389/fimmu.2021.733961

**Published:** 2021-10-05

**Authors:** Lena Margarethe Wulfken, Jürgen Christian Becker, Rami Hayajneh, Annette Doris Wagner, Katrin Schaper-Gerhardt, Nina Flatt, Imke Grimmelmann, Ralf Gutzmer

**Affiliations:** ^1^ Skin Cancer Center Hannover, Department of Dermatology and Allergy, Hannover Medical School, Hannover, Germany; ^2^ Translational Skin Cancer Research (TSCR), University Duisburg-Essen, Essen, Germany; ^3^ German Cancer Consortium (DKTK) & German Cancer Research Center (DKFZ), Heidelberg, Germany; ^4^ Department of Dermatology, University Hospital Essen, Essen, Germany; ^5^ Center for Medical Biology, Essen, Germany; ^6^ Clinic for Kidney and Hypertension Diseases, Hannover Medical School, Hannover, Germany; ^7^ Department of Dermatology, Ruhr University Bochum, Minden, Germany

**Keywords:** case report, B cell depletion, immune checkpoint blockade, CD20, melanoma

## Abstract

**Introduction:**

Checkpoint-Inhibition (CPI) with PD-1- and PD-L1-inhibitors is a well-established therapy for advanced stage melanoma patients. CPI mainly acts *via* T-lymphocytes. However, recent literature suggests also a role for B cells modulating its efficacy and tolerability of CPI.

**Case Report:**

We report a 48-year-old female patient with metastatic melanoma affecting brain, lung, skin and lymph nodes. A preexisting granulomatosis with polyangiitis was treated with rituximab over five years prior to the diagnosis of melanoma, resulting in a complete depletion of B cells both in peripheral blood as well as the tumor tissue. In the absence of the mutation of the proto-oncogene b-raf, treatment with the PD-1 inhibitor nivolumab was initiated. This therapy was well tolerated and resulted in a deep partial response, which is ongoing for 14+ months. Flow cytometric analysis of peripheral blood mononuclear cells revealed 15% IL-10 producing and 14% CD24 and CD38 double positive regulatory B cells.

**Conclusion:**

The exceptional clinical response to nivolumab monotherapy in our patient with depleted B cells sheds a new light on the relevance of B cells in the modulation of immune responses to melanoma. Obviously, B cells were not required for the efficacy of CPI in our patient. Moreover, the depletion of regulatory B cells may have improved efficacy of CPI.

## Introduction

Immune Checkpoint-Inhibition (CPI) is a well-established therapy for advanced or metastatic melanoma patients by blocking programmed death 1 (PD-1), programmed death ligand 1 (PD-L1) or cytotoxic T-lymphocyte-associated antigen 4 (CTLA-4). PD-1, a T cell co-inhibitory receptor, is expressed on activated T cells, B cells and myeloid cells. It binds to its ligands PD-L1 and PD-L2 resulting in inhibition of T cell proliferation, as well as inhibition of interferon-gamma, tumor necrosis factor-alpha and interleukin-2 production. Moreover, the survival of T cells is reduced. Recent evidence suggests also a role for B cells in the efficacy of CPI, in particular if present in tertiary lymphoid structures (1, 2). Tertiary lymphoid structures are amongst ectopic lymphoid organs resembling germinal center formation and emerge next to chronic inflammation such as tumorous tissue (3).

Granulomatosis with polyangiitis (GPA) is a systemic vasculitis affecting small- and medium-sized blood vessels and is often associated with the presence of anti-neutrophil cytoplasmic antibodies (ANCA) and proteinase-3 (PR3). Clinical manifestations involve primarily the upper and lower respiratory tract, however renal and cardiac involvement can occur. Immunosuppressive therapy is the standard for treatment. Since B cells are of high importance for autoimmune diseases by producing autoantibodies and pro-inflammatory cytokines, the anti CD20 antibody rituximab shows to be effective in GPA (4, 5). Rituximab depletes all CD20+ B cells without affecting bone marrow B cells or plasma cells. Therefore, rituximab is approved for induction therapy as well as maintenance treatment in GPA (5).

## Case Report

We report a case of a woman being first diagnosed with a high-risk melanoma plantar left in August 2016 with a Breslow thickness of 4,5mm. Excisional surgery and sentinel lymph-node extirpation was performed: TNM classification 8^th^ Edition (American Joint Classification of Cancer, AJCC): pT4a pN0 (sentinel lymph node negative) cM0 (no metastasis in clinical and radiologic examinations). Proto-oncogenes B-RAF, NRAS and cKIT were not mutated. In May 2019, she suffered from progressive disease with asymptomatic metastases in brain, lungs, lymph nodes and skin (stage IV M1d, AJCC). Serum levels of lactate dehydrogenase and S100 were within normal limits.

**Figure 1:**
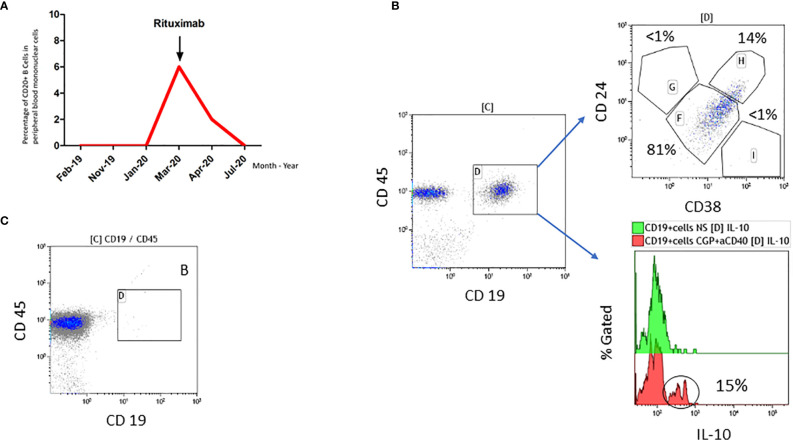
**(A)** Time course of B cell population in percent and rituximab administration in our patient. **(B)** Analysis of peripheral blood mononuclear cells obtained in March 2020 once B cells were detectable. 14% of the CD19+ B cells expressed the Breg markers CD24 and CD38 (Gate H), and 15% of CD19+ B cells produced IL-10 after stimulation with CpG and aCD40L. Gate F (mature/naïve/transitional B cells), Gate G (memory B cells), Gate I (plasma-like cells). **(C)** Analysis of peripheral blood mononuclear cells obtained two weeks after application of rituximab in April 2020 demonstrating complete absence of CD19+ B cells.

In addition, since 1996 the patient suffered from c-ANCA and PR3 positive granulomatosis with polyangiitis with an involvement of eyes (intraorbital granuloma), naso-pharyngeal mucosa (nasal secretions, saddle nose, subglottal stenosis), lungs and joints (arthritis with pain and swelling). Previous immunosuppressive therapies, i.e. cyclophosphamide, glucocorticoids, methotrexate, infliximab, azathioprine and mycophenolate, were not able to control the disease sufficiently. Since 2014, the GPA is well controlled with rituximab, an anti CD20 antibody, resulting in a complete depletion of B cells in peripheral blood and complete absence of clinical symptoms (**Figure 1**). Rituximab administrations are triggered by B cell counts in peripheral blood and/or clinical manifestations such as tachycardia, internal unrest and increased naso-oro-pharyngeal exsudation. The last rituximab administration before the diagnosis of metastatic melanoma was in January 2019 (**Figure 1A**).

**Figure 2:**
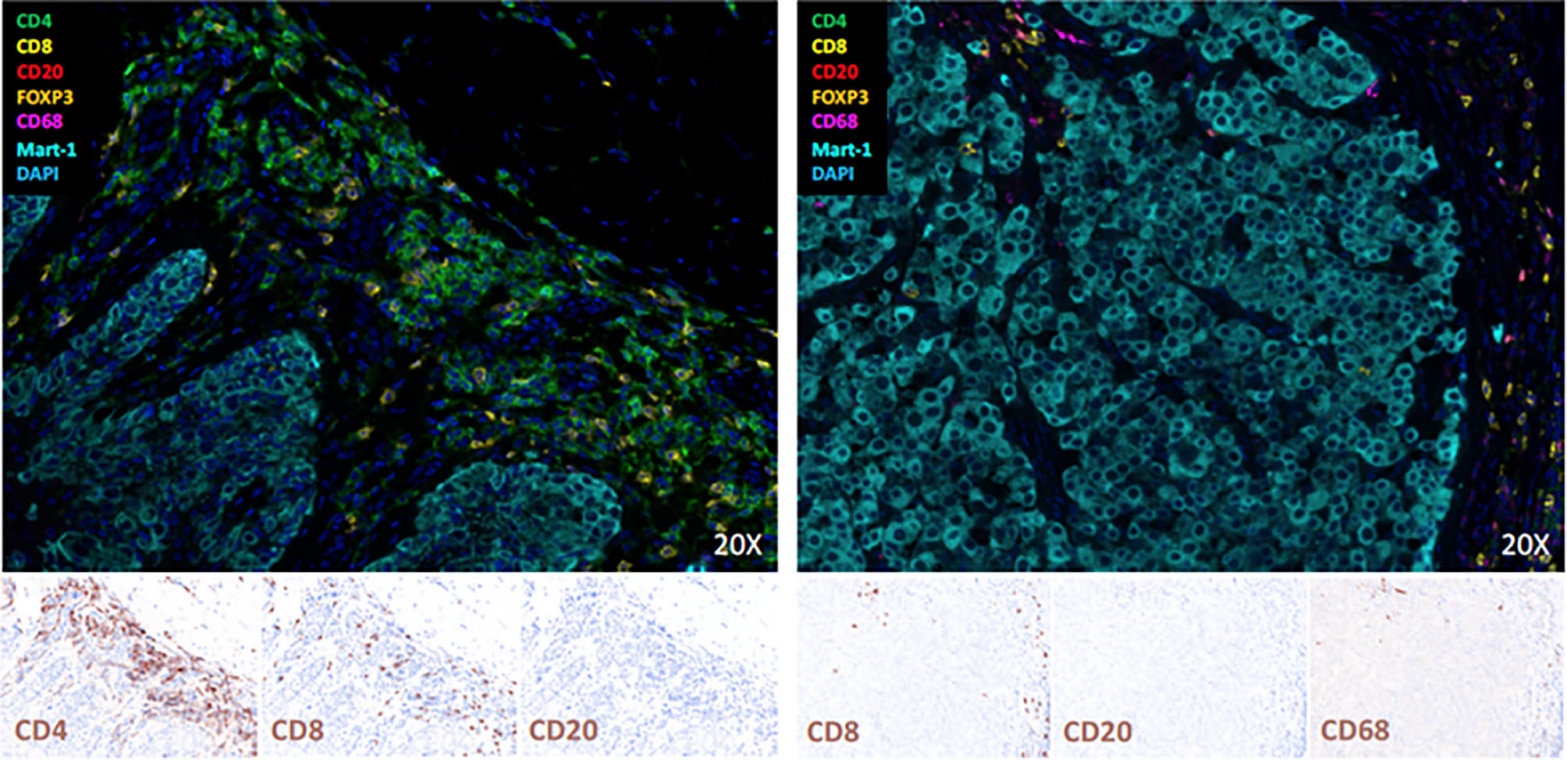
Multiplex Immunofluorescence staining and Immunohistochemistry of the primary melanoma (left) and a cutaneous metastases (right). B-cells are completely absent, and there is a peritumoral inflammatory infiltrate consisting primarily of CD4 and CD8 T-cells in the primary tumor and few CD8 T-cells and CD68 positive myeloid cells in the metastasis.

We analyzed tissue of the primary melanoma (from August 2016) and of a cutaneous metastasis (from May 2019) by multicolor immunofluorescence (Multiplex fluorescence immunohistochemistry staining *via* Opal-technology (AKOYA BIOSCIENCES, OP7TL4001KT) with Anti-CD4, -CD8, -CD20, FOXP3, Anti-CD68, and Melan-A according to manufacturer instruction). Results showed that B cells were completely absent and there was a peritumoral inflammatory infiltrate consisting primarily of CD4+ and CD8+ T cells in the primary tumor, whereas we found no CD4+ T cells, but only a few CD8+ T cells and CD68+ myeloid cells in the metastasis (**Figure 2**).

After discussion in the interdisciplinary board treatment with PD-1 inhibitor Nivolumab 240mg once every two weeks (q2w) was initiated for metastatic melanoma in June 2019. Tolerability was excellent with no side effects, and partial response with a reduction of size of all metastases, including brain metastases, and no new metastases was observed and is still ongoing for more than 14 months, the best possible clinical outcome for the patient.

**Figure 3:**
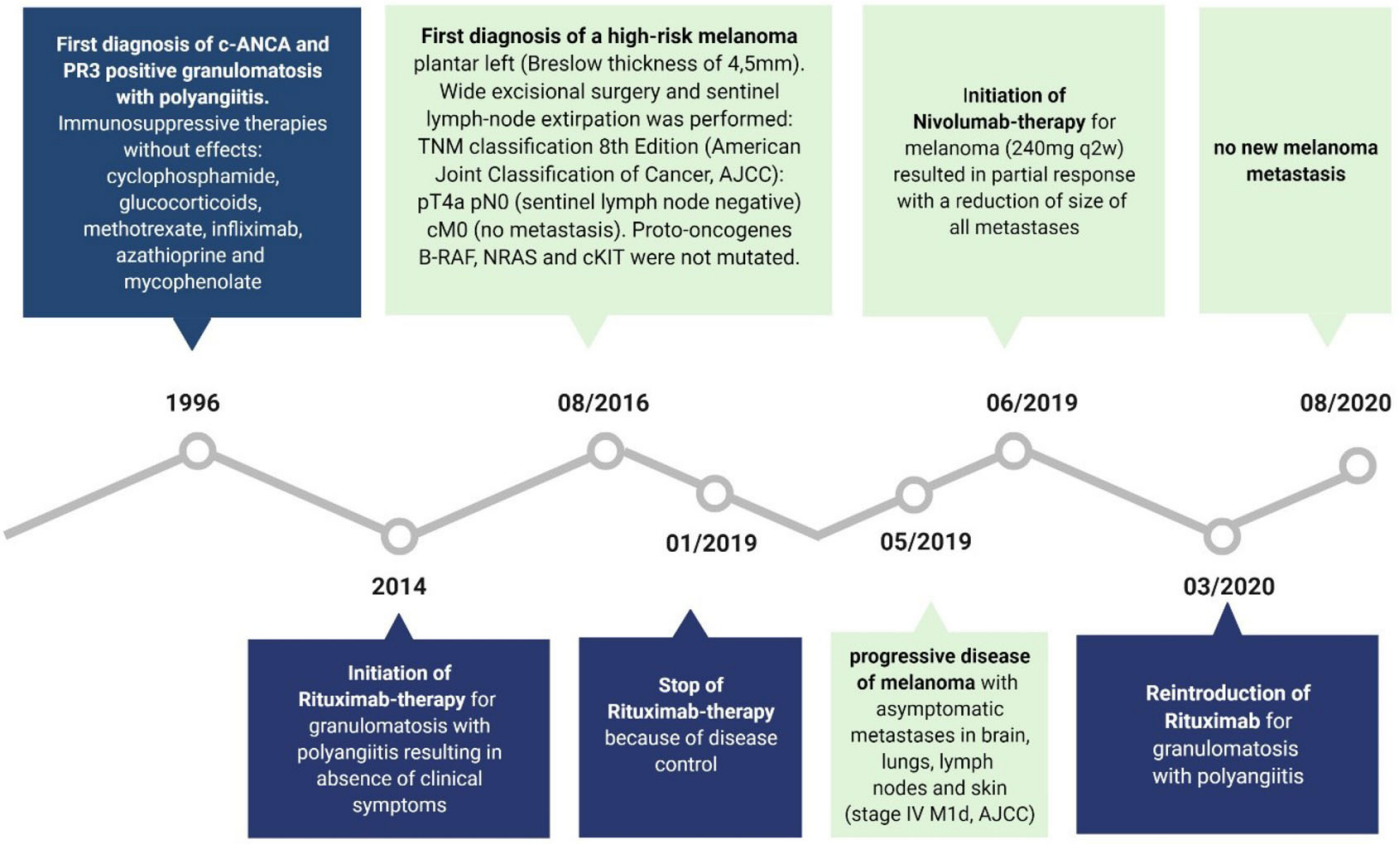
The medical timeline is demonstrated. Created with BioRender.com.

With regard to rituximab therapy, from January 2019 until March 2020, no administration was necessary; in March 2020, rituximab was applied again due to increasing B cells. Flow cytometric analyses of B cells before rituximab treatment in March 2020 revealed a high percentage of CD19+ cells expressing regulatory B cell (Breg) markers CD24 and CD38 (14%) as well as producing IL-10 (15%) (**Figure 1B**). For comparison: Zaimoku et al. (6) showed a frequency of around 3% of CD24+ CD38+ B cells in a healthy control group, while two other studies reported a frequency of 4% and 7%, respectively in CD19+IL10+ cells in healthy control groups (7, 8). After rituximab application in April 2020, no CD20 positive B cells were detectable anymore (**Figure 1C**). **Figure 3** demonstrates the timeline.

## Discussion

The role of B cells on the efficacy of CPI are under intense discussion, while clinical data are not available yet. Three scenarios are possible:

First, B cells have no effect. This is suggested by the observation that the density of B cells in tumor specimen did not correlate with response or overall survival in 40 melanoma patients treated with CPI (9). The authors extended this study to a preclinical model, demonstrating no differences in tumor growth or response to CPI in this murine melanoma model in correlation to the eradication of B cells (9).

Secondly, B cells could have a positive effect on CPI efficacy. This notion is suggested by recent reports that the presence of B-cells within tumors -particularly if arranged in tertiary lymphoid structures- were associated with an improved clinical outcome in patients treated with CPI for melanoma (1, 2). Notably, a similar observation has been previously reported in a preclinical model on immunotherapy of murine melanoma (10).

Thirdly, B cells could be associated with a negative effect in CPI. In a murine melanoma model intratumoral Breg accelerated tumor growth depending on IL-10 production of Breg (11). Recent studies indicate that the clinical efficacy of CPI is reduced in patients suffering from melanoma, cutaneous squamous cell carcinoma or Merkel cell carcinoma with B cell chronic lymphatic leukemia (B-CLL) (12). Neoplastic B cells of B-CLL often are characterized by a Breg-like phenotype (12). Another study demonstrated an impairment of BRAF-targeted therapy by B-cell derived IGF-1, which could be overcome by rituximab treatment in some patients (4).

With regard to GPA and CPI, recent literature shows only a few case reports. One patient showed no flare of an asymptomatic, ANCA-negative GPA well treated with methotrexate before initiating pembrolizumab for non-small cell lung cancer (13). Another patient treated with pembrolizumab for advanced non-small cell lung lancer primarily led to a flare of a preexisting, clinically silent, GPA.

Thus, CPI can trigger a flare in GPA, and it is unclear in which patients this flare occurs. In our patient, a beginning flare was associated with an increase with B cell counts but not with CPI.

In the here presented case, B cells were clearly not required for a deep and durable clinical response to CPI in this advanced melanoma. It is even tempting to speculate that the response to CPI was improved due to the absence of (regulatory) B cells.

However, since B cells are a very heterogenous subgroup of lymphocytes, interindividual differences on the effect of B cells on CPI efficacy have to be expected. Therefore, further research is necessary to define the role of B cells – or B-cell subsets – with respect to the outcome of CPI in cancer patients.

## Patient Perspective

Since suffering from GPA in 1996 with an involvement of eyes, naso-pharyngeal mucosa, lungs and joints without being improved by immunosuppressive therapies such as i.e. cyclophosphamide or glucocorticoids, the GPA is now well controlled with rituximab. Luckily there is a complete absence of clinical symptoms resulting in a high health-related quality of life. Moreover the metastatic melanoma shows a partial response with a reduction of size of all metastases due to Nivolumab administrations, which are well tolerated without showing any side effects.

## Data Availability Statement

The original contributions presented in the study are included in the article/supplementary material. Further inquiries can be directed to the corresponding author.

## Ethics Statement

Written informed consent was obtained from the individual(s) for the publication of any potentially identifiable images or data included in this article.

## Author Contributions

LW acquired and interpreted the data, and drafted the manuscript. JB, RH, AW, KS-G, NF, and IG acquired and interpreted the data. RG designed the work, acquired and interpreted the data, and drafted the manuscript. All authors contributed to the article and approved the submitted version.

## Funding

This study was supported by grants from the Claudia-von-Schilling Foundation and the BMS Immunooncology Foundation.

## Conflict of Interest

LW: Advice for MSD Sharp & Dohme GmbH, support of meeting participation by Novartis. JB: speaker’s bureau honoraria from Amgen, Pfizer, MerckSerono, Recordati and Sanofi; consultant/advisory board member/DSMB member for Boehringer Ingelheim, eTheRNA, InProTher, MerckSerono, Pfizer, 4SC, and Sanofi/Regeneron. His group receives research grants from Bristol-Myers Squibb, Merck Serono, HTG, IQVIA, and Alcedis. RG: personal fees and non-financial support from Bristol Myers Squibb, personal fees and non-financial support from Roche, grants, personal fees and non-financial support from Merck Serono, grants, personal fees and non-financial support from Amgen, personal fees and non-financial support from Pierre Fabre, personal fees and nonfinancial support from Sanofi Regeneron, personal fees from Merck Sharp and Dohme, grants, personal fees and non-financial support from Novartis, personal fees from Almirall Hermal, grants and personal fees from Pfizer, personal fees and grant from SUN Pharma, personal fees from 4SC, grant from Johnson&Johnson. IG: honoraria from Roche, MSD, BMS, Novartis. Consultant or Advisory Role for Roche, BMS, Novartis. Research Funding from Novartis, Pfizer.

The remaining authors declare that the research was conducted in the absence of any commercial or financial relationships that could be construed as a potential conflict of interest.

## Publisher’s Note

All claims expressed in this article are solely those of the authors and do not necessarily represent those of their affiliated organizations, or those of the publisher, the editors and the reviewers. Any product that may be evaluated in this article, or claim that may be made by its manufacturer, is not guaranteed or endorsed by the publisher.

## Abbreviations

CPI, Checkpoint-Inhibition; PD1, Programmed death 1; PD-L1, Programmed cell death ligand 1; IL-10, Interleukin-10; CD, Cluster of differentiation; CTLA-4, cytotoxic T-lymphocyte-associated antigen 4; GPA, Granulomatosis with polyangiitis; B-RAF, Proto-oncogene B-RAF; NRAS, Proto-oncogene Neuroblastoma-RAS; cKIT, Proto-oncogene Tyrosinkinase-KIT; ANCA, anti-neutrophil cytoplasmic antibodies; PR3, Proteinase-3; AJCC, American Joint Committee on Cancer; Breg, regulatory B cell; q2w, once in two weeks.
